# The Efficacy of Dental Caries Telediagnosis Using Smartphone: A Diagnostic Study in Geriatric Patients

**DOI:** 10.7759/cureus.33256

**Published:** 2023-01-02

**Authors:** Pragya Pandey, Neha Jasrasaria, Rhythm Bains, Abhishek Singh, Manish Manar, Abhinav Kumar

**Affiliations:** 1 Conservative Dentistry and Endodontics, King George's Medical University, Lucknow, IND; 2 Community Medicine and Public Health, King George's Medical University, Lucknow, IND

**Keywords:** teledentistry, telemedicine, smartphone, geriatric dentistry, dental caries

## Abstract

Background and aim

The major barrier to oral health care for geriatrics is access to dental care. Teledentistry is the field of dentistry that uses telecommunication with digital imaging for the exchange of valid information for diagnosis, treatment, and continuing dental education, enabling some means of approach to oral health care. This study aimed to assess the accuracy of diagnosing dental caries in the older age group by examining intraoral photographs taken by smartphone camera compared with standard clinical dental examination.

Methods and material

A calibrated dentist examined 18 patients aged 60-75 for six variables: sound tooth, decayed tooth, filled tooth with and without caries, missing tooth, and the presence of prosthesis. Intraoral photographs of each patient were taken using a smartphone. The photographs were then sent to six different examiners through WhatsApp. The accuracy of the diagnosis was measured by applying Cohen's kappa, sensitivity, and specificity. Fleiss' kappa was used to measure the agreement index using the software R (R Foundation for Statistical Computing, Vienna, Austria) version 4.2.1.

Results

The overall agreement between the tele-examiners and the control was measured, with the kappa scores ranging from 0.897 to 0.921, and the mean kappa score was 0.909. There was high specificity and sensitivity in the value observed. The agreement index, measured through Fleiss' kappa for six teeth conditions, reported the perfect agreement (0.867) among six examiners.

Conclusions

Under the limitations of the study, teledentistry using a mobile phone has acceptable accuracy for diagnosing caries in the older age group compared to a standard clinical dental examination.

## Introduction

Teledentistry is the use of electronic information and telecommunications for oral health care and oral health promotions. It is a branch of telemedicine that has evolved from combining communication technology and dentistry. The different modalities of teledentistry are synchronous live videos, asynchronous teledentistry, remote patient monitoring, and mobile health (mHealth) care services [1]. Mobile smartphones are excellent teledentistry tools as they are readily available, user-friendly, and cheap [2].

Nowadays, dental photography is a standard element of daily practice and plays an important role in dental diagnosis, consultations, and referrals. Teledentistry models using smartphone have shown high acceptability [2]. Few recent studies have shown the considerable reliability of mobile phone photographs in diagnosing dental caries in children. A study done by Subbalekshmi et al. on Indian children reported effective screening for early childhood caries (ECC) with digital images being taken in a school setting [3]. However, there is sparse information on the use of teledentistry in screening oral health and dental caries for the elderly group.

The proportion of the geriatric population is multiplying worldwide, and the need for medical and oral health services for this population is increasing. According to the United Nations, the number of senior citizens has risen from 5% of the population in 1950 to 10% in 2016, which is estimated to grow to 19% by 2050. This means that one in every five Indians will be 60 years or older [4]. Older adults tend to have multiple chronic medical conditions such as heart disease, hypertension, and diabetes. These systemic diseases also affect oral health, increasing the need for managing conditions such as periodontal disease, coronal caries, root caries, xerostomia, and other related oral health problems. Dental caries is an infectious disease that negatively impacts the quality of life of the elderly [5]. Oral afflictions such as pain, discomfort, and difficulty in chewing lead to poor nutrition, further deteriorating general health. An increase in physical and mental disabilities also makes older adults more vulnerable to poor oral health [6].

Many new materials and techniques have emerged to cater to the preventive, curative, and other dental needs of the elderly, but constant vigilance for caries is a must in this population. Unfortunately, as elderly patients are usually dependent, routine dental screening is not ensured. In a study by Fish-Parcham et al., access to dental health services is one of the major issues in providing older adults with oral healthcare services [7]. Telediagnosis and telescreening might be possible solutions to these challenges as they avoid unnecessary visits to the dental office. Additionally, it is a viable solution to address the need for underserved populations such as those in geographically remote, marginalized, or less developed areas and those who are homebound and living in elderly care institutions [4]. Telehealth services are essential in a country such as India with a poor doctor-to-patient ratio and huge inequity in providing healthcare service [8].

Telehealth is more important in scenarios such as the COVID-19 pandemic, where older people are at a higher risk of developing severe illness if they contract the disease [9]. Therefore, under the Ayushman Bharat Scheme of the Government of India, the Ministry of Health and Family Welfare started the scheme of e-Sanjeevani in November 2019. e-Sanjeevani outpatient department (OPD) is a doctor-to-patient telemedicine system through which anyone can seek medical consultation by connecting to the doctor through audio and video. It is also known as the National Teleconsultation Service, which aims to provide healthcare services to patients in their homes [10]. However, telemedicine and teledentistry are relatively new subjects and need to be studied for their potential and limitations.

Ben-Omran et al. in 2021 did a scoping review on the use of teledentistry in facilitating oral health for older adults. They reported the promising potential of telehealth services in older adults for various oral healthcare services, i.e., consultations, oral health promotions, screening, and referrals. However, none of the included studies screened for the diagnosis of dental caries in older adults using teledentistry models [11]. Given the exigent need for telecare for the older age group, this study aims to evaluate the reliability of smartphone in diagnosing dental caries in Indian geriatric patients.

## Materials and methods

Ethical considerations

Data was collected after obtaining ethical approval from the Institutional Ethics Committee of King George's Medical University (XI-PGTSC-II B BDS-S/P8). All participants were given information sheets and were enrolled after taking prior consent. Signed informed consent for the use of intraoral photographs was also obtained. Photos were unnamed and only showed the participants' dentition. For each study participant, a separate file with a unique identification number was maintained to ensure confidentiality. The examining dentists were blinded to the true identity of the patient.

Sample size and study setting

This study was done on adults aged 60-75 years visiting the OPD of the Department of Conservative Dentistry and Endodontics of King George's Medical University. A review of past OPD attendance revealed that on average, 10 patients between the age group of 60 and 75 years visited the OPD of the Department of Conservative Dentistry and Endodontics per day. A total of about 500 patients are expected to visit the OPD during the 60 days of data collection. For 80% sensitivity and 80% specificity, the sample size required for this study was 18, considering the 50% level of significance and the 80% power of the study. A total of 18 cases among six examiners yielded 108 comparisons. The reference value of sensitivity and specificity and the number of examiners to be calibrated are based on the existing published literature by AlShaya et al. who evaluated the reliability of mobile phone teledentistry in dental diagnosis in mixed dentition [12].

Inclusion and exclusion criteria

The patients aged 60-75 years who visited the OPD of the Department of Conservative Dentistry and Endodontics of King George's Medical University for routine checkups or any other dental complaint were included in the study. The patients who did not give consent to participate, were edentulous, or had limited mouth opening were excluded from the study. In addition, the patients with severe fluorosis, hypoplasia, or any serious systemic disease were also excluded.

Study protocol

The patients fulfilling inclusion criteria were recruited by simple random sampling using a random number generator. The patients were examined by an experienced dentist (endodontist). The diagnosis of dental caries was made by following the World Health Organization (WHO) oral health assessment form for adults (version 2013) [13]. The control examiner, doing the standard clinical examination, was priorly self-calibrated by performing repeat examinations on 10 adults aged between 60 and 75 years. Six experienced dentists (endodontist) with similar experience were calibrated with the benchmark examiner. These six dentists served as the "telescreening group." The telescreening group made their diagnoses by evaluating the images sent to them through WhatsApp messenger.

Clinical dental and teledentistry examination

The clinical examination was done using the World Health Organization (WHO) category I examination criteria using professional light and mirror. The diagnosis was made for sound teeth, missing teeth, decayed teeth, filled teeth with and without caries, and any prosthesis if present.

**Figure 1:**
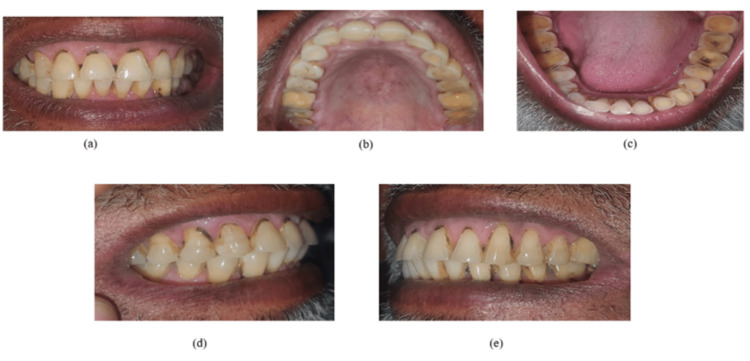
Intraoral photographs (a) Front view, (b) upper occlusal view, (c) lower occlusal view, (d) right lateral view, and (e) left lateral view

As shown in Figure 1 (front, upper occlusal, lower occlusal, right lateral, and left lateral), five pictures of a patient were taken on a mobile phone camera. For all captured photos, similar settings were used. The dentist (principal investigator) was responsible for taking the pictures. Priorly, he received hands-on training for capturing clear images of the mouth. The pictures obtained were stored in a folder with a unique identification number for each patient on Google Drive, an online cloud platform.

The pictures were then shared with the telescreening group using WhatsApp, a social media application that uses the internet to send images. Next, the benchmark dentist and the telescreening group charted their findings on the adult version of the WHO oral health assessment form [13]. After that, the patient was advised a treatment plan as per the need.

Smartphone specifications

The camera of a Samsung A53 (Samsung Electronics Co., Ltd., Suwon, South Korea) with quad 64 MP (ƒ/1.8 aperture) was used. The phone has optical image stabilization with up to 10× digital zoom. In order to ensure standardization, the same smartphone was used for taking all the photographs.

Statistical analysis

A total of 18 patients resulted in 90 intraoral photographs and 108 comparisons. A total of 3456 teeth were examined and compared by the control examiner. A p value of ≤0.05 was considered to be statistically significant. The accuracy of the teledentistry examination was determined by calculating the sensitivity, specificity, positive predictive value (PPV), and negative predicative value (NPV). The diagnosis of the dental examination was reported as either true positive (TP), false positive (FP), true negative (TN), or false negative (FN). The calculations of specificity and sensitivity were done using the following formulas: Sensitivity = TP/TP + FN and Specificity = TN/TN + FP [14]. For each participant, the diagnosis reported by the standard clinical dental examination was considered as the correct diagnosis, i.e., either true positive or true negative. If the diagnosis reported by tele-examination was contrary to the clinical examination, it was considered false positive or false negative as the case may be.

The PPV indicates how often a positive diagnosis by tele-examination represents a true positive. The NPV signifies how often a negative test reported by tele-examination represents a true negative. The following formulas were used to report the PPV and NPV: PPV = TP/TP + FP and NPV = TN/TN + FN.

The agreement between the clinical examination and tele-examination was calculated using the following formula: Agreement (TP + TN/total (TP + FP + FN + TN)) and Cohen's kappa. Kappa result was interpreted as follows: values of ≤0 as no agreement and 0.01-0.20 as none to slight, 0.21-0.40 as fair, 0.41-0.60 as moderate, 0.61-0.80 as substantial, and 0.81-1.00 as almost perfect agreement [15]. Fleiss' kappa was used to measure the agreement index using software R (R Foundation for Statistical Computing, Vienna, Austria) version 4.2.1.

## Results

The study population comprised 18 adults. The mean age of females and males was 63.6 years (SD ±3.21) and 65.5 years (SD ±4.46), respectively. The results of the Shapiro-Wilk test (W = 0.92 and p = 0.18) showed that the age variable was normally distributed. Consequently, there was no significant difference between the mean age of males and females (Table 1).

**Table 1:** Overall inter-examiner variability

Teeth condition	Fleiss' kappa	Z-value	P-value
Sound	0.830	76.1	0.000
Decayed	0.749	68.7	0.000
Filled with caries	0.354	32.5	0.000
Filled with no caries	0.449	41.2	0.000
Missing	0.991	90.9	0.000
Prosthesis	0.903	82.8	0.000
Total	0.867	195	0.000

The patients were classified according to their chief complaint. "Tooth decay" (17.71%) was the most observed dental condition among examined patients with dental problems, followed by "missing teeth" (13.02%) (Table 2).

**Table 2:** Mean age of the study population

Gender	Number (N)	Mean	Standard deviation (SD)	T-value	P-value
Female	7	63.6	3.21	-1.09	0.292
Male	11	65.5	4.46		

A total of 3456 teeth were examined and compared by the control examiner. The overall agreement between the examiners (using teledentistry) and the control was observed, with kappa scores ranging from 0.897 to 0.921. The mean kappa score was 0.909. There was high specificity and sensitivity in the value observed. The results revealed that positive predictive values and negative predictive values were almost constant across all the examiners (Table 3). The balanced accuracy for all the examiners was found to be greater than 90% and ranged from 94% to 97%.

**Table 3:** Total number of teeth conditions and readings for each score (as per standard clinical examination)

Teeth condition	Number (N)	Percentage (%)
Sound	362	62.85
Decayed	102	17.71
Filled with caries	4	0.69
Filled with no caries	13	2.26
Missing	75	13.02
Prosthesis	20	3.47
Total	576	100

The agreement index, measured through Fleiss' kappa for six teeth conditions, i.e., filled with caries, filled with no caries, missing, sound, decayed, and prosthesis, reported the perfect agreement (0.867) among six examiners. The individual values for Fleiss' kappa are summarized in Table 4.

**Table 4:** Overall agreement between the tele-examiner group and the control

Parameters	Examiner 1	Examiner 2	Examiner 3	Examiner 4	Examiner 5	Examiner 6
Sensitivity	91.8%	93.0%	92.8%	93.5%	93.4%	91.9%
Specificity	98.2%	98.4%	98.5%	98.6%	98.4%	98.3%
Positive predictive value (PPV)	91.1%	92.1%	92.7%	93.2%	92.1%	91.5%
Negative predictive value (NPV)	98.4%	98.6%	98.6%	98.7%	98.7%	98.4%
Balanced accuracy	94.9%	95.7%	95.7%	96.1%	95.8%	98.4%
Kappa score	0.897	0.911	0.913	0.921	0.913	0.900

## Discussion

This study shows that the combination of digital images from smartphone camera and store and forward method for telecommunication offers a valid and reliable means of screening dental caries.

Six variables assessed were sound teeth, decayed teeth, missing teeth, filled (with/without caries) teeth, and the presence or absence of prosthesis. An overall high level of agreement was observed among the two groups, tele-examination and clinical dental examination, reporting high sensitivity (>91%), high specificity (>98%), and perfect agreement (kappa value of 0.897-0.921).

The utilization of diagnostic tests in patient care settings is evidence-based. As teledentistry is a relatively new topic, it needs more validation regarding its efficacy, accuracy, and application in a few arenas [16]. Although many studies have evaluated its validity in screening oral lesions, oral cancer, impacted third molars, and early childhood caries, there are virtually no studies on telediagnosing dental caries in older Indian adults [3,12].

In a recent study by AlShaya et al. in 2022, a sensitivity of 95% and specificity of 94.3% for pictures taken by a dentist and a sensitivity of 98.3% and specificity of 91.4% for pictures taken by a non-dentist were reported in primary teeth. Similarly, 80.8% and 88.5% sensitivity and 94.1% and 96.1% specificity were observed for permanent teeth for pictures taken by a dentist and non-dentist, respectively [17]. Amável et al. did a study on Portuguese kindergarten children aged 4-6 years. He used store and forward photograph as a teledentistry model for diagnosing dental caries. The results showed high sensitivity and high specificity values for the photographic method, which ranged from 94% to 100% and 52% to 100%, respectively. The average PPV and NPV were 80% and 97%, respectively [18].

Sensitivity and specificity are essential indicators of the accuracy and appropriateness of a diagnostic tool [14]. The sensitivity of any diagnostic test determines true positive findings, i.e., the extent to which telescreening can correctly identify participants who have dental caries. Contrarily, the specificity is the extent to which the telescreening can correctly identify participants who do not have dental caries [14]. In most previous studies, the sensitivity of the photographic assessments ranged from 43% to 100%, and the specificity ranged from 52% to 100%.

Higher sensitivity, specificity, and agreement found in the present study could be due to many reasons. Firstly, earlier studies targeted younger age group: preschool or school-going children who tend to be relatively less cooperative in a dental office and have smaller-size mouth opening. Secondly, the intraoral photos in this study were captured by the dentist who initially received hands-on training in dental photography. These factors helped in capturing better-quality images.

The present study used a smartphone as a teledentistry tool to capture intraoral pictures. The study shows that the use of affordable alternatives such as smartphone camera and store and forward method offers a reliable means for the screening of dental caries in older adults. Smartphones are readily available and user-friendly, and the area of interest can be easily captured by zooming in or out. Digital intraoral images were forwarded using WhatsApp, a popular social media platform and a real-time communication channel [19]. Until the last decade, the applicability of teledentistry was limited by the usage of low-resolution cameras [20] and limited information transfer [21]. The current study used a better-resolution mobile camera to review patient photographs. A high level of agreement (kappa value of >0.8) observed in the present study between the standard clinical diagnosis and telediagnosis of dental caries could largely be attributed to this.

In geriatric patients, considering the SiSta classification of Mount and Hume, caries is mainly encountered at tooth neck at all stages of progression and less seen on the occlusal site and proximal sites. This may be one reason for making carious lesion more conspicuous in geriatric patients [22]. Moreover, the variables such as missing teeth and the absence of prosthesis can easily be identified in images leading to better predictive values and higher specificity.

Boye et al. in 2012 used 50 extracted human permanent teeth to compare photographic and visual assessment of occlusal caries. Their findings reported that the median sensitivity for the photographic and the visual assessments were 81.3% and 65.5%, respectively [23]. They used histology as the reference standard, which could be one of the reasons for lower sensitivity and specificity seen in their study.

It is well established that there is an underestimation of caries occurrence when radiographs are not used along with clinical examination particularly with interproximal caries. As reported in a study by Hopcraft and Morgan, the prevalence and decayed, missing, and filled surface (DMFS) score showed a significant increase with the addition of radiography along with clinical examination across all age groups (p < 0.001) [24]. Hence, the present study did not estimate the caries prevalence in the study sample.

The level of agreement among the examiners is a critical issue observed in most investigations [12]. Cohen's kappa value observed in the study was comparable to AlShaya et al.'s study, which observed a kappa of 0.89 between tele-examination and clinical examination [12].

The specificity observed in the current study was similar to that of Boye et al. [25]. The present study results demonstrated superior sensitivity (>90%) and specificity (>98%) than the investigations conducted in the recent decade [25-27]. We believe that the higher experience of examiners and good-quality images played a vital role in improving the sensitivity, specificity, and high level of agreement.

Torres-Pereira et al. applied the principles of teledentistry in the remote diagnosis of oral lesions by sharing the images through a mail-based system [27]. The study results successfully demonstrated an accurate remote diagnosis of teeth deformations. Remote dental diagnosis is the need of the hour in rural India, where dental facilities are limited.

The study had certain limitations. Firstly, considering the applicability of teledentistry, the sample population should have been recruited from a limited facility or an elderly care center. This would have allowed the testing of teledentistry in remote locations for distant diagnosis. In addition, the involvement of a non-dentist in taking pictures would have simulated the real scenario and helped achieve more specific results. Secondly, the patient data was available to the examiners, which might have allowed them to identify age-specific patterns. Patient details, as well as the oral condition, could have been blinded. Lastly, screening was limited to the presence or absence of caries; however, the site of the lesion could be an added variable. Some of the shortcomings of this remote diagnosis might be issues with the internet especially in marginalized areas and the confidentiality of the data. However, teledentistry is not technique-sensitive, nor does it require a rigorous learning curve; still, the basic training and implementation need more reassurance.

## Conclusions

Dental caries remains the primary cause of tooth loss in older age. As the new generation of subjects over 65 years of age is increasingly dentulous, hence more exposed to the risk of caries, the current study focused on the telescreening of dental caries in geriatrics. Unfortunately, most aged patients are unable to visit a dentist due to multiple physical, mental, and social challenges. This has delegated the use of innovative technical skills, i.e., teledentistry, as an acceptable tool for health approach for this vulnerable age group.

Teledentistry has a wide scope and is applicable in all specialties of dentistry. Despite certain limitations, the present study shows acceptable reliability for the remote screening of dental caries in older adults with the use of intraoral pictures using smartphone camera and store and forward method, compared to standard clinical examination. Further, larger and good-quality research should focus on comparing different modalities of teledentistry with face-to-face methods to avail its best application for geriatric dental care. Evidence-based standard operating protocols (SOPs) for teledentistry models must be established before adopting this technology fully in the healthcare system. Furthermore, this study might intrigue young minds to improve existing technology and develop innovations pertaining to geriatric patient care and comfort.
